# Racial Disparity in HCV Demographics and Treatment Between Interferon Era (2002–2003) and Direct Acting Anti-viral Era (2019)

**DOI:** 10.7759/cureus.36643

**Published:** 2023-03-24

**Authors:** Paul Naylor, Ria Minawala, Katherine Wong, Murray N Ehrinpreis, Milton Mutchnick

**Affiliations:** 1 Gastroenterology, Wayne State University School of Medicine, Detroit, USA; 2 Internal Medicine, Wayne State University School of Medicine, Detroit, USA

**Keywords:** viral genotype, cirrhosis, fibrosis, hepatitis, interferon, direct acting anti-virals, linkage to care, epidemiology, racial diversity, hepatitis c virus

## Abstract

Introduction

Direct-acting antiviral (DAA) treatment increased the sustained viral response (SVR) rate of patients with the hepatitis C virus (HCV) and eliminated response disparities between African American (AA) and non-AA patients seen with interferon (IFN). The aim of this study was to compare 2019 HCV patients (DAA era) to patients from January 1, 2002 and December 31, 2003 (IFN era) in our predominantly AA clinic population.

Methods

We extracted data on 585 HCV patients seen in 2019 (DAA era) and compared them to 402 patients seen in the IFN era.

Results

Most HCV patients were born between 1945 and 1965, but in the DAA era more younger patients were identified. Non-AA patients in both eras were less likely to be infected with genotype 1 compared to AA (95% vs 54%, P<0.001). Fibrosis was not increased in the DAA Era as compared to the IFN era as assessed either by serum-based assays (APRI, FIB-4) or transient elastography (FibroScan) (DAA era) vs biopsy (IFN era). More patients were treated in 2019 compared to 2002-2003 (159/585=27% vs 5/402=1%). For untreated patients, subsequent treatment within one year of the initial visit was low and similar in both eras (35%).

Conclusion

There continues to be a need to screen patients born between 1945 and 1965 for HCV as well as to identify increasing numbers of patients below this age cohort. Even though current therapies are oral, highly effective, and can be 8-12 weeks in duration, significant numbers of patients were not treated within a year of first visit.

## Introduction

Worldwide, approximately 58 million people are infected with chronic hepatitis C virus (HCV) with an incidence of 1.5 million new infections every year [1]. In the US, HCV remains the most common bloodborne illness with approximately 2.4 million people infected and with a twofold higher prevalence in African Americans (AA) as compared to non-AA [2,3]. Since patients with HCV remain asymptomatic until they develop sequelae of cirrhosis, various screening protocols have been recommended.

Highly effective and safe direct-acting antivirals (DAA) for HCV increased the sustained viral response (SVR) rate (>95%) and eliminated the treatment response disparities between AA and non-AA patients seen previously with interferon (IFN) based therapy [4-7]. It was expected that these highly effective therapies when combined with screening would detect, treat, and reduce HCV infection. The initial screening protocol was the U.S. Preventive Services Task Force recommendation to screen for HCV in individuals born between 1945 and 1965 (age cohort; 54-74 years of age in 2019). This recommendation was subsequently updated in 2020 to include one-time testing of all adults [8]. This was due to increasing rates of infection in younger adults presumably due to the “opioid” epidemic [9,10]. Unfortunately, both identification and linkage to treatment in HCV patients remain low [11,12].

The aim of our study was to compare 2019 HCV patients (DAA era) in our predominantly AA urban Detroit clinics to patients from January 1, 2002 to December 31, 2003 (IFN era) to evaluate racial disparity demographic trends and treatment status. We hypothesized an increase in successfully treated patients due to the advent of DAAs and an increase in the number of patients being treated after their first visit.

Portions of this article were previously presented as a meeting abstract at the 2021 American College of Gastroenterology on October 25,2021. The abstract was also published in the meeting proceedings as Ria Minawala, Katherine Wong, Paul Nayor, Murray Ehrinpreis, Milton Mutchnick S1243: Comparing Racial Disparity in HCV Demographics and Treatment Between 2002-2003 and 2019 The American Journal of Gastroenterology: October 2021 - Volume 116 - Issue - p S573 doi: 10.14309/01.ajg.0000778504.91333.84 [13].

## Materials and methods

Using electronic medical records, we identified 585 HCV patients seen in 2019 (DAA era) and compared them to 402 patients seen between January 1, 2002 and December 31, 2003 (IFN era) in the Wayne State University School of Medicine clinics. All patients had confirmed HCV infection and at least one visit to either gastroenterology (GI) or Infectious disease clinic (ID) in the time period. Continuity of care was defined as the initiation of treatment within one year of the first visit and successful treatment was defined as sustained virus response (SVR) at 12 weeks after ending treatment. Only 11 patients were seen in both time periods and for analysis, they were included in the 2019 patient population.

The Wayne State Institutional Review Board approved this study under a HIPPA waiver since the data was collected from the medical records and informed consent was not required. Data collected included demographics (gender, race (AA or non-AA), age at a clinic visit, Body Mass Index (BMI), past medical conditions, laboratory studies (HCV genotype, viral load, alanine transaminase (AST), aspartate transaminase (ALT) and platelets), imaging studies, and treatment history (dates of treatment, a medication used). Fibrosis was assessed directly by transient elastography (FibroScan) (2019) or biopsy (2002-2003) and as serum-based calculations of the AST Platelet Ratio Index (APRI) and Fibrosis-4 (FIB-4) scores.

Statistical analysis was performed using JMP software from Statistical Analysis Software (SAS). Numeric data were plotted and evaluated using ANOVA and character data was evaluated using Pearson's chi-square analysis. Statistically significant differences were defined as a p-value < 0.05.

## Results

There were 997 patients in total, with 402 seen in the IFN era (January 1, 2002 and December 31, 2003) and 585 seen in the DAA era (2019). Three times more patients were seen per year in the DAA era as compared to the IFN era. Only 11 patients were seen in both eras which were 17 years apart. Of these patients, the majority (nine of 11) were treated with DAA therapy prior to their 2019 visit. Of the nine DAA-treated patients, five were treatment-naïve with respect to the IFN era and four had failed IFN-Ribavirin prior to successful DAA treatment. The other two repeat patients seen in 2019 were not treated within one year after their initial appointment. For this study, repeat patients are included in the DAA-era patient population.

**Figure 1:**
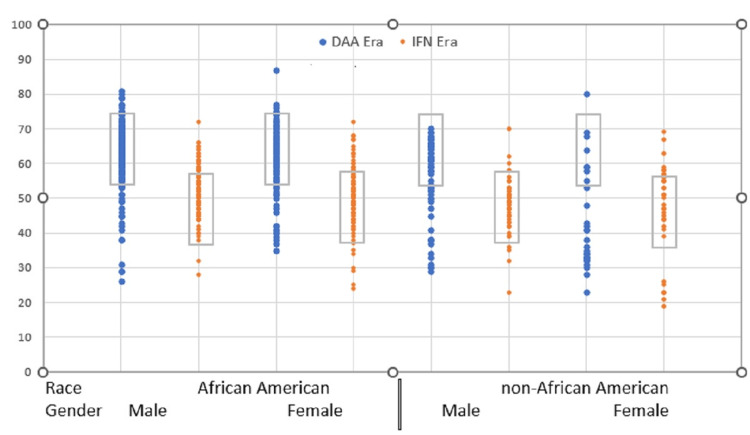
Age of HCV patients seen in the DAA era or the IFN Era by race and gender. Age at first visit for each era (Direct Acting Antiviral Era [DAA, 2019 patients] or IFN Era [Interferon, 2002 and 2003 patients]) is plotted as a function of race and gender.  AA patients were older than non-AA patients in both eras (63 vs 51 years in DAA era; p= 0.0001) and (51 vs 48 years; p=0.0001 in IFN era). The rectangular bars indicate screening age cohort patients born between 1945 and 1965 (DAA era = 74-54 years and IFN era = 57-37 years).

Figure 1 plots the age distribution of individual patients for each era by gender and race. Most of the patients were AA (85%) and there were slightly more males (66%). The average age of male and female AA patients and male non-AA patients was older in the 2019 era, whereas non-AA females were younger. While the IFN era patients were typically identified by either risk assessment or abnormal serum liver values, the patients in the DAA era were also identified due to the screening recommendation for patients born between 1945 and 1965. Thus, Figure 1 also uses boxes to identify patients whose birth date puts them in the “baby boomer” cohort screening group. Younger patients who are not included in the 1945-1965 screening group are clearly more likely to be in the recent DAA era. While the older age patients in both eras are in the 1945-1965 cohort, there are significant numbers of individuals in the DAA era (as compared to the INF era) of both races and gender who are younger than the age cohort.

**Figure 2:**
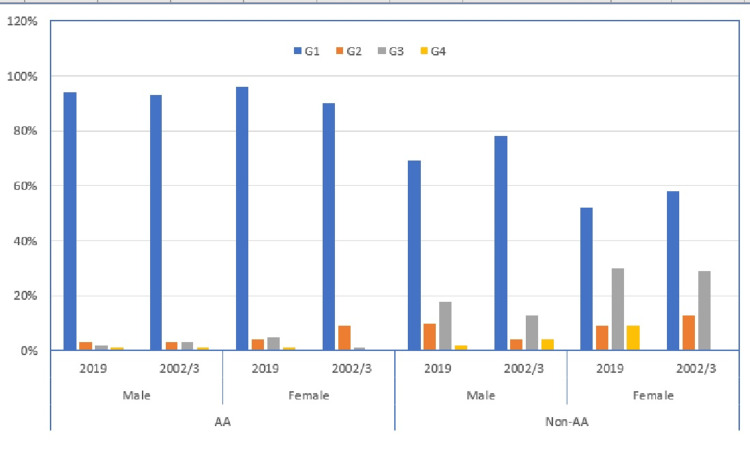
Genotype of HCV virus by race and gender. The percentage of patients with each genotype are plotted by era, race and gender. Non-African American (AA) patients of either gender in both eras (DAA era for 2019 HCV patients and IFN era for 2002 and 2003) were less likely to be infected with genotype 1 compared to AA patients of either gender (95% vs 54%, p<0.001).

Non-AA patients in both eras were less likely to be infected with genotype 1 compared to AA (95% vs 54%, P<0.001: figure 2). This disparity in racial distribution of genotype continues to be present and yet unexplained. However, unlike the IFN era, the development of pan genotypic DAA treatment means this observation has minimal impact on therapeutic treatment decisions.

Table 1 compares liver-relevant serum assays for the untreated patients by race, gender, and era. Viral load greater than 2 million copies/ml was more likely to be present in 2019 patients but changes in assay methodology are a likely confounder given the considerable time between the two measurements. Also as shown in Table 1, even though the viral load may be higher, the liver function tests were improved as defined by a lower AST and ALT and a higher platelet count in the DAA era patients as compared to the IFN era.

**Table 1:** HCV characteristics between 2 eras 17 years apart by race and gender. AA, African Americans'; non-AA, all other races. BMI, Body Mass Index; AST, aspartate transaminase; ALT, alanine transaminase; viral load, HCV virus in international units per liter with greater than 2x10^6^ being defined as high. Direct acting antiviral (DAA) Era are HCV patients seen in 2019; Interferon (IFN) era are HCV patients seen in 2002 and 2003. Significance is defined using ANOVA analysis with * indicating p<0.01 and ** indicating p<0.001.

	Number of Patients	BMI	Albumin	Platelet	AST	ALT	Hi Viral Load (>2X10^6^)
Male AA DAA era	327	27	4.1	228**	44**	42*	40%**
Male AA IFN era	184	27	4.0	204	70	101	19%
Female AA DAA era	173	30	3.8	233	45**	37**	41%**
Female AA IFN era	129	31	3.7	231	66	66	15%
Male non-AA DAA era	55	26	4.2	211	51**	60**	45%**
Male non-AA IFN era	53	28	4.2	196	87	101	23%
Female non-AA DAA era	30	29	4.2	257	41**	49	29%**
Female non-AA-IFN era	36	26	4.0	221	68	70	9%

Fibrosis as defined by APRI and Fib-4 was lower in 2019 but only APRI was statistically significant (Table 2; p=0.0001). This decline in fibrosis was true for both races. In contrast, cirrhosis by ultrasound was higher the DAA Era and this was true for both races. (Table 2). Although biopsies were rarely performed in 2019, transient elastography (FibroScan) data was available to provide a direct measurement of fibrosis. When the presence of significant fibrosis (F2-F4) by FibroScan was compared to biopsy result in 2002-2003, there was no significant difference between the two eras.

**Table 2:** Fibrosis by race and era using four methods. AA, African Americans; non-AA, all other races. Direct Acting Anti-Viral (DAA) Era, HCV patients seen in 2019; Interferon (IFN) era, HCV patients seen in 2002 and 2003. Significance (p<0.05) for differences between the two eras is defined using Pearson Chi-Square analysis. *Cirrhosis was defined by Ultrasound in both eras, **Biopsy was used to define fibrosis in the 2002 and 2003 HCV patients and or transient elastography (FibroScan) to define fibrosis in the 2019 HCV patients. ***AST to Platelet Ratio Index (APRI) is calculated as (AST/40)/Platelet). ****Fibrosis-4 (FIB-4) is calculated as (Age x AST)/(Platelet x Sqrt ALT).

Overall	Cirrhosis^#^	Fibrosis ( F2-F4)^## ^	APR^###^	Fib-4^####^
DAA Era	10%	56%	23%	15%
IFN Era	2%	50%	45%	18%
Significance (Pearson)	P=0.0001	P=0.15	P=0.0001	P=0.17
Race				
AA DAA Era	10%	45%	23%	19%
AA IFN Era	2%	52%	44%	17%
non-AA DAA Era	15%	35%	32%	32%
non-AA IFN Era	0%	46%	48%	22%
Significance (Pearson Chi-Square)	P=0.0001	P=0.29	P=0.0001	P=0.033

More patients of both race categories were treated prior to their visit in the DAA era as compared to the IFN era (159/585=27% vs 5/402=1%; Table 3). AA patients in the IFN era were also less likely to have been treated prior to the visit (0.43% vs 5% p=0.0017). For untreated patients, subsequent treatment within one year of the initial visit was similar in both eras (35%; Table 3).

**Table 3:** Patient treatment status by race and era. AA, African Americans and non-AA, all other races. Direct Acting Anti-Viral (DAA) Era are HCV patients seen in 2019; Interferon (IFN) Era are HCV patients seen in 2002 and 2003. Significance (p<0.05) for differences between the two eras is defined using Pearson Chi-Square analysis.

Race Era	Number of Patients/Total Patients	% of Patients	Significance vs Era
Treated before visit (includes in progress)			
AA DAA Era	138/500	27%	
AA IFN-Era	1/313	0.23%	p=0.0001
non-AA DAA Era	21/85	25%	
non-AA IFN Era	4/89	5%	p=0.0001
All DAA Era	159/585	27%	
All IFN Era	5/402	1%	P=0.0001
Treated after visit (within 1 year)			
AA DAA Era	129/366	35%	
AA IFN Era	96/312	31%	NS (p>0.05)
non-AA DAA Era	24/66	36%	
non-AA IFN Era	35/86	40%	NS(p>0.05)
All DAA Era	153/432	35%	
All IFN Era	131/398	33%	NS(p>0.05)

Unlike the earlier interferon era where over 95% of HCV patients were treated by Gastroenterology physicians, in 2019 significant numbers of patients (24%) were treated by non-GI physicians in infections disease clinics (Table 4). Also, unlike the IFN Era, the available therapies were considerably wider (Table 4). 

**Table 4:** Physician group treating HCV patients in the direct acting antiviral era. Direct Acting Anti-Viral (DAA) Era are HCV patients seen in 2019.

Direct Acting Antiviral (DAA)	Number	%	Number	%
	Gastroenterology Clinics		Infections Disease Clinics	
Mavret (glecaprevir/pibrentasvir)	109	47%	34	47%
Harvoni (ledipasvir/sofosbuvir)	74	32%	16	22%
Epclusa (sofosbuvir/velpatasvir)	36	16%	15	21%
Zapatier (elbasvir/grazoprevir)	7	3%	7	10%
Other	6	3%	1	1%

## Discussion

Although current therapies for HCV are well-tolerated, highly effective, and have a shorter duration of treatment (DAA era) as compared to older interferon-based treatments (IFN era), a significant number of our clinic patients were not treated within a year of the first visit during the DAA era (65%). During the IFN era, the low treatment rate was due primarily to the low rate of SVR in AA patients, the long treatment period, patient hesitancy to be treated given significant side effects from the medication, and insurance denial. The low treatment rate in both the AA and non-AA population in the DAA era was unexpected. While most of our patients had Medicaid, they were seen in 2019 when Michigan Medicaid had modified its policy to cover the treatment of all HCV patients regardless of the degree of fibrosis. The primary issue was that most patients failed to return to the clinic within 1 year of their diagnosis and assessment for treatment. This was possibly due to multiple factors, including lower health literacy, one failure to appreciate the long-term risk for cirrhosis and HCC, access to transportation to clinic visits, and other personal or extenuating circumstances in our patient population. The hypothesis that treating patients at the first visit would increase treatment rates, remains to be evaluated. The literature on this issue also confirms low linkage to care especially with respect to the type of sites seeing the patients, the type of insurance, and poor follow up including an inability to contact the patients [11,12].

The increased age of AA and non-AA male patients during the DAA era is consistent with the aging of the baby boomer cohort and was expected as these patients are identified by screening and entering treatment discussions with providers. More concerning was the increase in patients with HCV who are younger than the older age cohort, especially female patients. This observation argues strongly for the one-time HCV testing recommendations [8]. The impact of the opioid epidemic on the increase in younger populations being infected with HCV cannot be determined from this study [9,10]. The continued appearance of older patients in the clinic also argues for an increase in efforts to target treatment and follow-up efforts since they may represent a cohort with the gradual evolution of the liver disease and at significant risk. This is especially important given the solid evidence that even patients with advanced liver disease benefit from treatment with respect to the progression of the disease [14-17].

There was some discordance when evaluating for fibrosis and cirrhosis in our DAA era population as compared to the Interferon era. The possibility that ultrasound methodology improved over this period and that patients were being screened and identified earlier could be a confounding issue that cannot be resolved. The higher rates of cirrhosis based on ultrasound imaging but lower levels of fibrosis on serum-based calculations highlight the well-known discrepancies between the methods of evaluation with respect to the identification of cirrhosis. When comparing biopsy in the early era vs FibroScan in the recent era, despite the reported concordance of the two methods, there was no significant difference. This observation is tempered by the fact that not all patients underwent biopsy in the IFN era and a similar situation was found for FibroScan in the DAA era. These observations further support the fact that clinicians should not depend on a single test for the evaluation and diagnosis of fibrosis and cirrhosis, but rather a combination of history and physical exam findings, imaging, and laboratory-based studies.

During the IFN era, all patients with HCV were treated only by a GI provider. However, during the DAA era, patients were being treated by both GI and ID providers. The decision with respect to who would provide treatment is complex. While HIV and hepatitis B (HBV) co-infection may play a role in the choice of referral, it is also possible that primary care providers who are performing the screening may have a preference with respect to referral. The method of HCV detection by primary care providers is also unclear since it could be risk-based, age cohort, or liver enzyme elevation. Future studies in our population could help elucidate these areas of uncertainty [14,18].

Our study did have some limitations. As a retrospective study, there are possibilities for selection, recall, or misclassification biases, as well as unidentified confounding factors. This is especially relevant with respect to cirrhosis comparison due not only to the lack of information regarding confounders such as body mass index, diabetes, alcohol use, and HBV coinfections but the fact that not all patients underwent biopsies or FibroScans and ultrasound is operator and equipment dependent with respect to assessing cirrhosis. , On the other hand, while patients were identified in the electronic medical records (EMR) using ICD-10 codes, all patients included in the study were confirmed to meet an inclusion criterion to provide an accurate diagnosis. Any risk of bias was tempered with carefully defined inclusion criteria, using clear definitions and cut-offs for the variables in the data collection, and ensuring that all data was collected in a similar way using a defined case reporting form. We also had a large sample size which lent to our findings being adequately powered. Lastly, our study was conducted at a single center with a population of primarily AA patients in an urban setting. Our findings may not be extrapolated to the general population, which is comprised of non-AA patients, private health insurance, and is in suburban or rural settings. Prospective studies with more diverse cohorts in various settings while always warranted are not readily performed.

While there are publications describing patients in each era, few address patients seen in the same clinical setting in two eras [19-23], and none that report on patients seen 17 years apart. Observations from these studies demonstrate an overall aging of the population along with an increase in the percentage of younger patients in the DAA era. Also inconclusive is the issue of whether the DAA population has increasing fibrosis and cirrhosis. The younger patients are less likely to have the advanced disease compared to the older patients who have been infected longer. Future studies should focus on re-examining our patient charts to evaluate reasons for low treatment rates during the DAA era. Also of interest is the issue of how patients are being identified and referred for treatment. This is important with respect to identifying and addressing any weaknesses in our system to improve treatment rates and patient follow-up.

## Conclusions

This study is unique in that it reports data on HCV patients seen in an inner city, predominately AA health care system over a 17-year period. The study demonstrates that although the population with HCV is aging, there is an increase in young patients who represent individuals infected with HCV in the recent era. This continued infection in younger patients while present in both genders and in AA and non-AA individuals, is consistent with the CDC recommendation that one time testing in all individual rather than using subjective risk-based screening for individuals regardless of whether they are in the age cohort. The data also demonstrates that AA individuals continue to have an as yet unexplained dominance of genotype 1 as compared to non-AA. With respect to disease status, patients seen in the 2019 era were not subjected to biopsy and although their fibrosis status was difficult to assess, treatment of all patients regardless of fibrosis is now possible with the current DAA therapies.

Despite that fact that current DAA therapies for HCV are well-tolerated, highly effective, and have a short duration of treatment, as many as 65% of recent era patients were not treated within a year of their first visit. Thus, this study demonstrates that additional efforts to identify the reason for this failure to provide treatment to patients who have had at least 1 visit to GI or ID are needed.
